# Preferential stimulation of melanocytes by M2 macrophages to produce melanin through vascular endothelial growth factor

**DOI:** 10.1038/s41598-022-08163-7

**Published:** 2022-04-19

**Authors:** Heeju Han, Yena Kim, Hyunkyung Mo, Si Hwa Choi, Kijun Lee, Yeri Alice Rim, Ji Hyeon Ju

**Affiliations:** 1 Catholic iPSC Research Center (CiRC), CiSTEM Laboratory, Department of Biomedicine & Health Sciences, College of Medicine, The Catholic University of Korea, Seoul, 06591 South Korea; 2 Department of Medical Life Sciences and Department of Biomedicine & Health Sciences, College of Medicine, The Catholic University of Korea, Seoul, 06591 South Korea; 3 Division of Rheumatology, Department of Internal Medicine, Seoul St. Mary’s Hospital, College of Medicine, The Catholic University of Korea, Seoul, 06591 South Korea

**Keywords:** Monocytes and macrophages, Induced pluripotent stem cells

## Abstract

Post-inflammatory hyperpigmentation is a skin discoloration process that occurs following an inflammatory response or wound. As the skin begins to heal, macrophages first exhibit a proinflammatory phenotype (M1) during the early stages of tissue repair and then transition to a pro-healing, anti-inflammatory phenotype (M2) in later stages. During this process, M1 macrophages remove invading bacteria and M2 macrophages remodel surrounding tissue; however, the relationship between macrophages and pigmentation is unclear. In this study, we examined the effect of macrophages on melanin pigmentation using human induced pluripotent stem cells. Functional melanocytes were differentiated from human induced pluripotent stem cells and named as hiMels. The generated hiMels were then individually cocultured with M1 and M2 macrophages. Melanin synthesis decreased in hiMels cocultured with M1 macrophages but significantly increased in hiMels cocultured with M2 macrophages. Moreover, the expression of vascular endothelial growth factor was increased in M2 cocultured media. Our findings suggest that M2 macrophages, and not M1 macrophages, induce hyperpigmentation in scarred areas of the skin during tissue repair.

## Introduction

Skin epidermal melanocytes are derived from the neural crest. Melanocytes play a major role in the production of melanin, which protects skin cells from damage caused by harmful ultraviolet radiation^1,2^. Melanocytes synthesize melanosomes (melanin-containing vesicles), which are transported to neighboring keratinocytes and confer color through a process known as pigmentation^3,4^. Defects in functional melanocytes lead to pigmentary disorders such as hyperpigmentation, hypopigmentation, vitiligo, and albinism. Genetic background, stress, inflammatory responses, and hormone may be responsible for these disorders^4–6^.

Melanocytes can be directly isolated from the epidermis. However, using melanocytes for research is challenging because of their limited quantity and poor proliferation. Studies of human induced pluripotent stem cell (hiPSC)-derived melanocytes are expected to improve the understanding of melanocyte-related diseases and melanocyte biology. It has been shown that hiPSCs can differentiate into the melanocyte lineage^7–11^. These human iPSC-derived melanocytes (hiMels) express representative melanocytic markers, such as MITF, TYR, and TYRP1 and can produce melanosomes and integrate into the reconstituted epidermis^12^. Moreover, hiMels show the ability to reconstruct hair follicles and the epidermis in vivo^9,13^*.*

Macrophages (MΦ) are differentiated from blood monocytes and exist as resident tissue-specific cells. Macrophages act as key innate immune cells by phagocytizing under pathological conditions. They also act as antigen-presenting cells in response to various pathogens, acting as a bridge between innate and adaptive immunity to re-establish homeostasis^14^. These cells are particularly active during inflammation and wound healing and can be classified into two major polarization states: classically activated type 1 macrophages (M1) and alternatively activated type 2 macrophages (M2)^15–17^. M1 macrophages are differentiated following injury or infection and produce proinflammatory cytokines, such as interleukin (IL)-1β, IL-6, and IL-12. In contrast, M2 macrophages are found during the wound healing phase, where they promote tissue remodeling by producing anti-inflammatory cytokines such as IL-10 and transforming growth factor (TGF)-β1 and angiogenesis-related cytokines such as vascular endothelial growth factor (VEGF) and CD31.

Post-inflammatory hyperpigmentation commonly occurs after cutaneous inflammation and can arise in all skin types^18^. Studies have shown that proinflammatory, anti-inflammatory, and angiogenic cytokines can affect melanocyte pigmentation^19,20^. Interferon (IFN)-γ blocks the maturation of melanosomes^21,22^, IL-1β downregulates *MITF* expression and inhibits melanocyte pigmentation^23^, and IL-6 decreases tyrosinase activity^24^. IL-18 increases tyrosinase activity^25^; IL-33 promotes MITF, TYR, TYRP-1, and TYRP-2 expression^26^; and VEGF stimulates melanin production in melanocytes^27^. However, the interaction between macrophages and pigmentation remains unclear.

In this study, we used an in vitro coculture system of macrophages and hiMels, focusing on the molecular effects of macrophage-secreted factors on melanocyte function. hiMels showed differing responses to the macrophage type. Melanin production was suppressed in the presence of M1 macrophages but increased in the presence of M2 macrophages. Moreover, we identified VEGF as the M2-derived factor that promotes melanin synthesis in hiMels. Our findings suggest that modulation of the immune environment regulates skin pigmentation.

## Results

### Differentiation of melanocytes from hiPSCs

**Figure 1:**
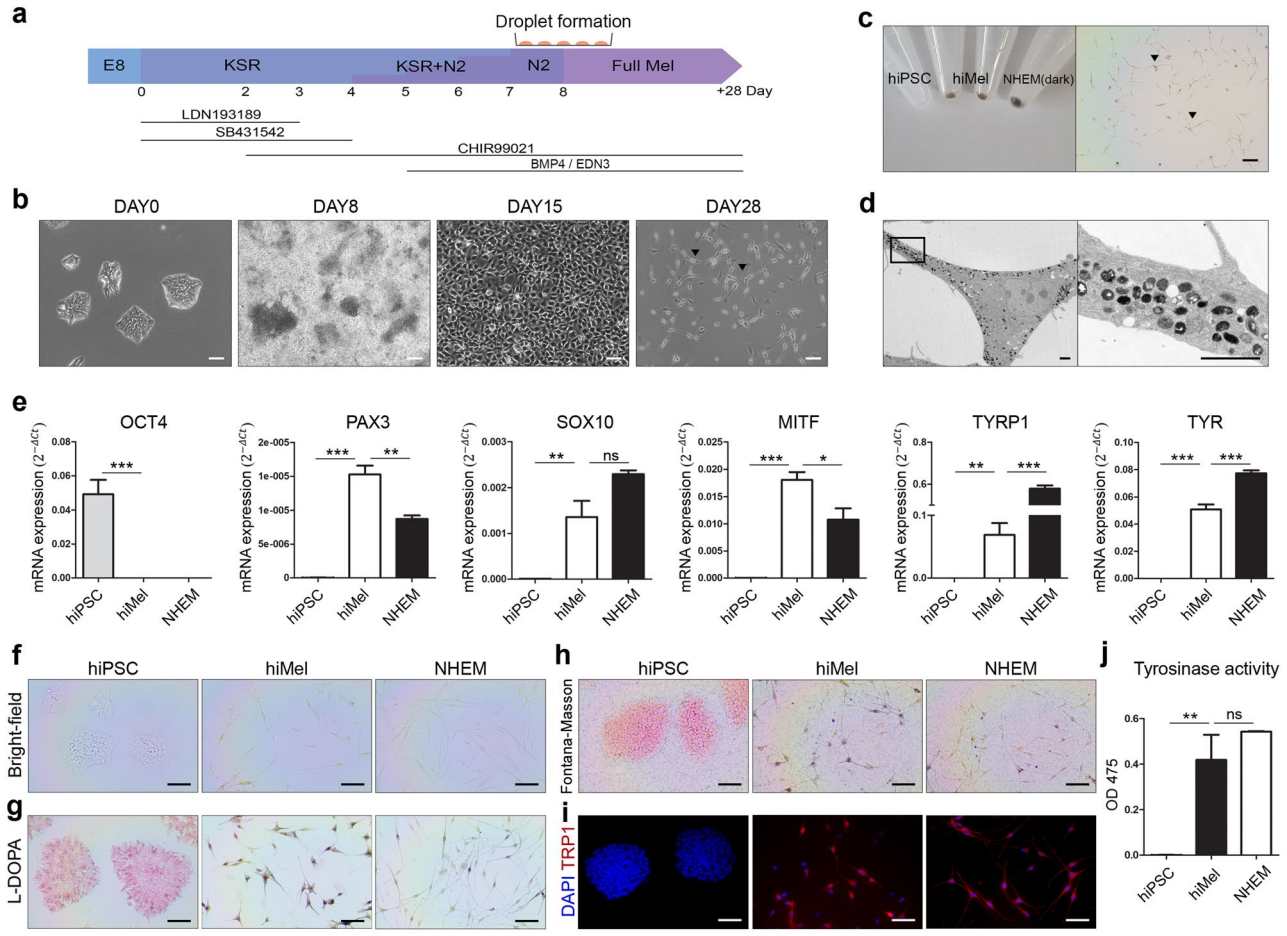
Differentiation of human induced pluripotent stem cell-derived melanocytes. (**a**) Schematic diagram of melanocyte differentiation from hiPSCs. (**b**) Morphology of cells at each stage of differentiation. Scale bar indicates 100 µm. (**c**) Image of day 28 cell pellets showing a darkly pigmented phenotype (left). Pigmented cells visualized under a bright-field microscope (right). Arrowheads indicate pigmented cells. Scale bar indicates 100 µm. (**d**) Transmission electron microscopy (TEM) image of melanosomes in hiMels. Scale bar indicates 2 µm. (**e**) Gene expression of the pluripotency marker *OCT4* and melanocyte markers *PAX3*, *SOX10*, *MITF*, *TYRP1*, and *TYR*. The relative gene expression values were calculated using the delta cycle-threshold (ΔCt) method. (**f**) Bright-field microscope images of hiPSCs, hiMels, and NHEMs. (**g**) L-DOPA staining of hiMels. Scale bar indicates 100 µm. (**h**) Fontana–Masson staining image of hiMels. (**i**) Immunocytochemical analysis of melanocyte marker TRP1 (red) and DAPI (blue). Scale bar indicates 100 µm. (**j**) Quantification of tyrosinase activity in hiMels. Data are expressed as the mean ± SEM. *p < 0.05, **p < 0.01, ***p < 0.001 indicate statistical significance. *ns* non-significant, *hiPSCs* human induced pluripotent stem cells, *hiMels* human hiPSCs derived melanocytes, *L-DOPA* 3,4-dihydroxyphenylalanine, *NHEM* normal human epidermal melanocyte.

To generate hiMels, we adapted protocols from other reports^7,28^. Peripheral blood mononuclear cell-derived hiPSC lines were used in this study. A schematic of the melanocyte differentiation protocol is shown in Fig. 1a. We observed morphological changes during differentiation (Fig. 1b). hiPSCs showed embryonic stem cell-like colonies during expansion. On day 28, the cell morphology changed to spindle-shaped cells with black pigmentation. Compared to hiPSC pellets, hiMel pellets exhibited a relatively dark shade on day 28 and were visible in the cells (Fig. 1c). Moreover, mature melanosomes were observed in the cytoplasm of hiMels (Fig. 1d). To confirm whether hiMels exhibited the characteristics of human epidermal melanocytes (NHEMs), we confirmed the expression of specific markers. NHEMs (darkly pigmented donor) were used as a positive control (Fig. 1e). The level of the pluripotency marker OCT4 was significantly reduced in differentiated cells and NHEMs. Positive expression of the melanocyte-specific markers PAX3, SOX10, MITF, TYRP1, and TYR was increased in hiMels. The differentiated hiMels were also positive for Fontana–Masson and L-DOPA staining but not for hiPSCs (Fig. 1f–h). Immunocytochemistry confirmed that expression of the mature melanocyte marker TRP1 was increased in hiMels compared to in hiPSCs (Fig. 1i). Tyrosinase activity measured at OD 475 nm indicated biosynthesis of melanin by hiMels (Fig. 1j). Together, these data demonstrate that hiPSCs can differentiate into functional melanocytes.

### Polarization of THP-1 monocytes into M1 and M2 macrophages

**Figure 2:**
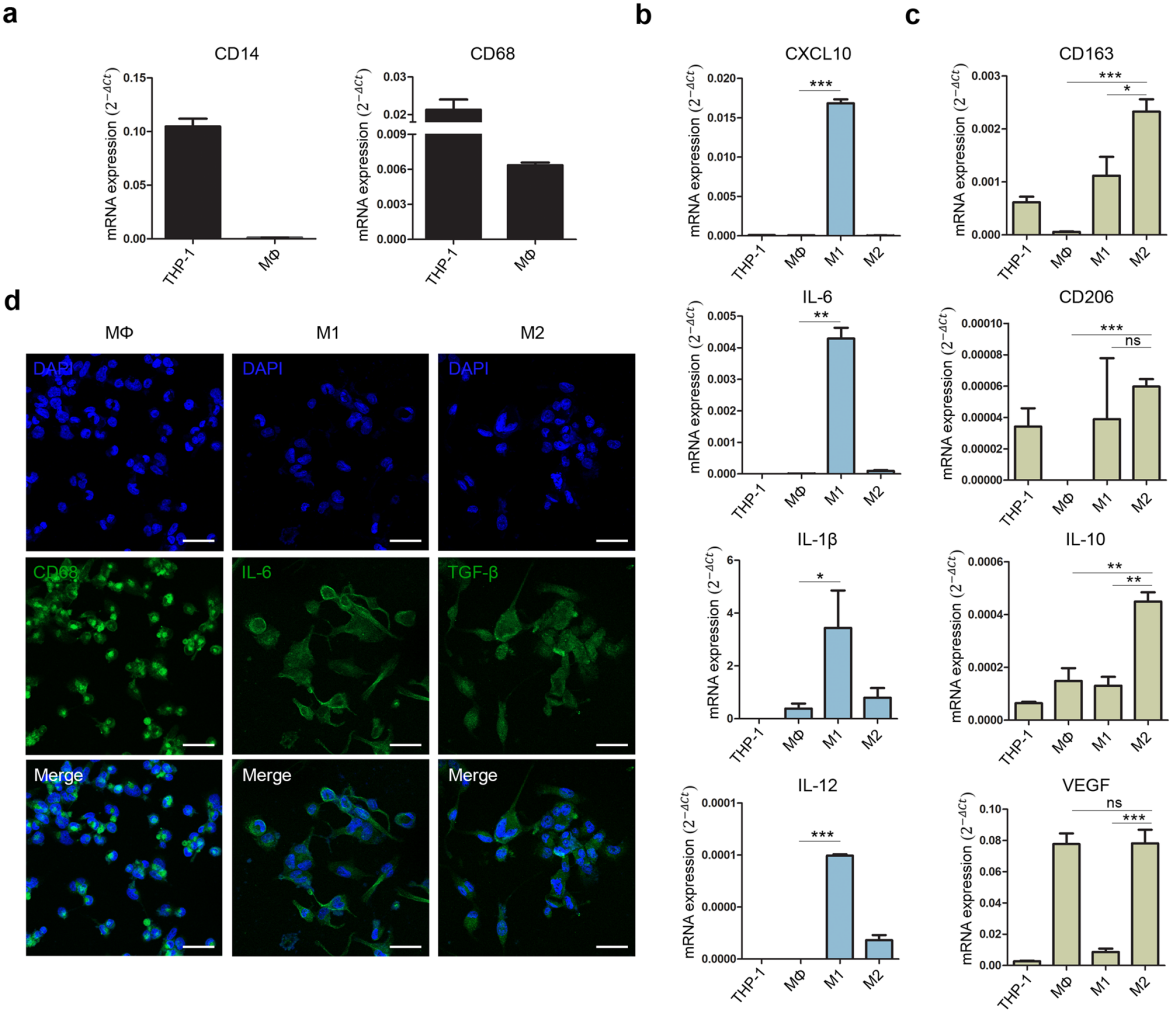
Polarization of M1 and M2 macrophages from THP-1. (**a**) Gene expression of the monocyte marker CD14 and monocyte/macrophage marker CD68 in macrophages differentiated from THP-1 monocytes. (**b**) Expression of the proinflammatory markers *CXCL10*, *IL-6*, *IL-1β*, and *IL-12*. (**c**) Expression of the anti- inflammatory markers *CD163*, *CD2206*, and *IL-10* and angiogenesis marker *VEGF.* The relative gene expression values were calculated using the delta cycle-threshold (ΔCt) method. Data are expressed as the mean ± SEM. *p < 0.05, **p < 0.01, ***p < 0.001 indicate statistical significance. (**d**) Immunofluorescence staining of CD68 (green), IL-6 (green), and TGF-β (green) and DAPI-stained nucleus (blue). Scale bar indicates 100 µm. *ns* non-significant, *MΦ* macrophage.

To induce M1 and M2 macrophages, THP-1 monocytes were treated with phorbol 12-myristate 13-acetate and differentiated into macrophages. The suspended THP-1 monocytes became adherent, and expression of the monocyte marker *CD14* decreased in macrophages compared to that in THP-1 monocytes (Fig. 2a). Expression of the monocyte/macrophage marker CD68 was higher in THP-1 monocytes than in differentiated macrophages. To polarize M1 macrophages, we treated macrophages with IFN-γ and LPS, as described previously^29^. The expression of proinflammatory markers, such as *CXCL10*, *IL-6*, *IL-1β*, and *IL-12*, was significantly increased in M1 macrophages (Fig. 2b). In contrast, M2 macrophages can be induced by treatment with IL-4 and IL-13^29^. Upregulated expression of the M2 macrophage markers *CD163*, *CD206*, *IL-10*, and *VEGF* was confirmed (Fig. 2c). Expression of each macrophage marker, CD68 (MΦ), IL-6 (M1), and TGF-β (M2), was positive in each cell (Fig. 2d). Based on these results, we obtained M1 and M2 macrophages that may mimic the inflammatory condition and anti-inflammatory environment in vitro.

### Coculture of hiMels with M1 or M2 macrophages

**Figure 3:**
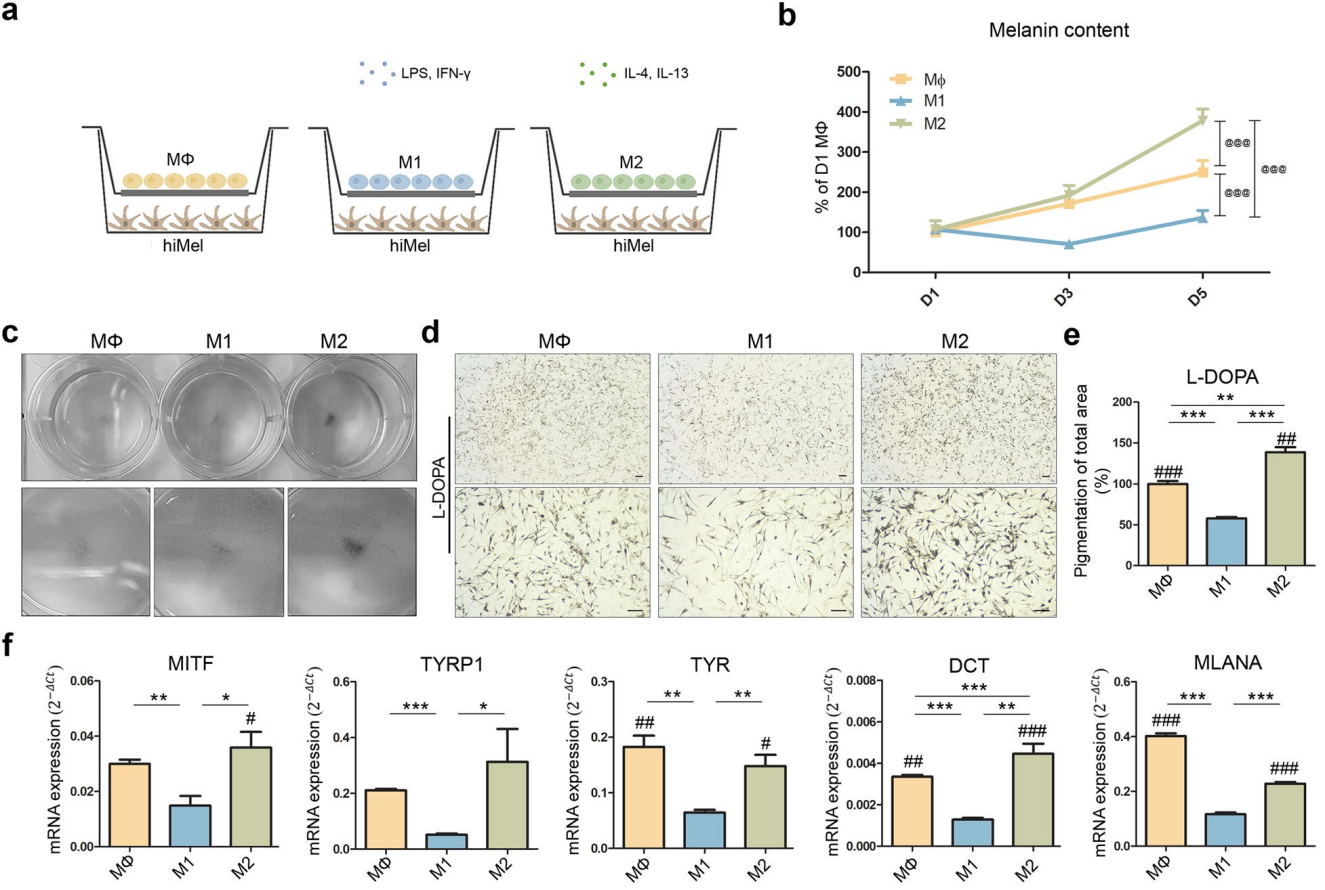
Coculture of hiMels with MΦ, M1, and M2 macrophages in vitro. (**a**) Schematic diagram showing coculture of hiMels and polarized macrophages. (**b**) Melanin content of cocultured hiMels measured on days 1, 3, and 5. (**c**) Whole image of the hiMel-culture plate after coculture (**d**) L-DOPA staining of cocultured hiMels on day 5. Scale bar indicates 100 µm. (**e**) Quantitative analysis of pigmented area in cocultured hiMels. (**f**) Gene expression of mature melanocyte markers *MITF*, *TYRP1*, *TYR*, *DCT* and *MLANA* in cocultured hiMels. The relative gene expression values were calculated using the delta cycle-threshold (ΔCt) method. ^@@@^*p* < 0.001 vs. M1 (two-way analysis of variance), *p < 0.05, **p < 0.01, ***p < 0.001 (*t*-test), ^#^*p* < 0.05, ^##^*p* < 0.01, ^###^*p* < 0.001 vs. M1 (one-way analysis of variance) indicate statistical significance. *MΦ* macrophage, *hiMels* human induced pluripotent stem cell (hiPSCs)-derived melanocytes, *L-DOPA* 3,4-dihydroxyphenylalanine.

To provide inflammatory and tissue repair environments, we cocultured hiMels with M1 or M2 macrophages (Fig. 3a). The cytokines secreted by the cells were predicted to be exchanged through the transwells. After 1, 3, and 5 days of coculture, melanin content was measured in cocultured hiMels (Fig. 3b). After 5 days, hiMels cocultured with M2 exhibited significantly increased melanin biosynthesis compared to hiMels cocultured with MΦ. However, hiMels cocultured with M1 showed no significant increase in the melanin content compared to the two groups. Figure 3c provides a whole image of the hiMel-coculture plate before staining. L-DOPA staining of cocultured hiMels showed a similar tendency as the measured melanin content (Fig. 3d). hiMels cocultured with M2 synthesized more melanin in the cytoplasm compared to hiMels cocultured with MΦ; however, hiMels cocultured with M1 synthesized less melanin compared to hiMels cocultured with MΦ (Fig. 3e). Expression of the melanocyte pigmentation markers *MITF*, *TYRP1*, *TYR*, *DCT*, and *MLANA* was quantified in cocultured hiMels (Fig. 3f). Marker expression was significantly lower in hiMels cocultured with M1 than in hiMels cocultured with MΦ. hiMels cocultured with M2 showed increased levels of pigmentation markers compared with hiMels cocultured with M1. These results confirm that the proinflammatory environment induced by M1 macrophages decreased melanin biosynthesis in hiMels. In contrast, melanin biosynthesis was increased in hiMels cocultured with M2, indicating that the tissue repair environment induces melanin biosynthesis. These findings suggest that skin pigmentation is affected by the in vivo environment, specifically by resident macrophages.

### Cytokine evaluation in coculture media of hiMels and macrophages

**Figure 4:**
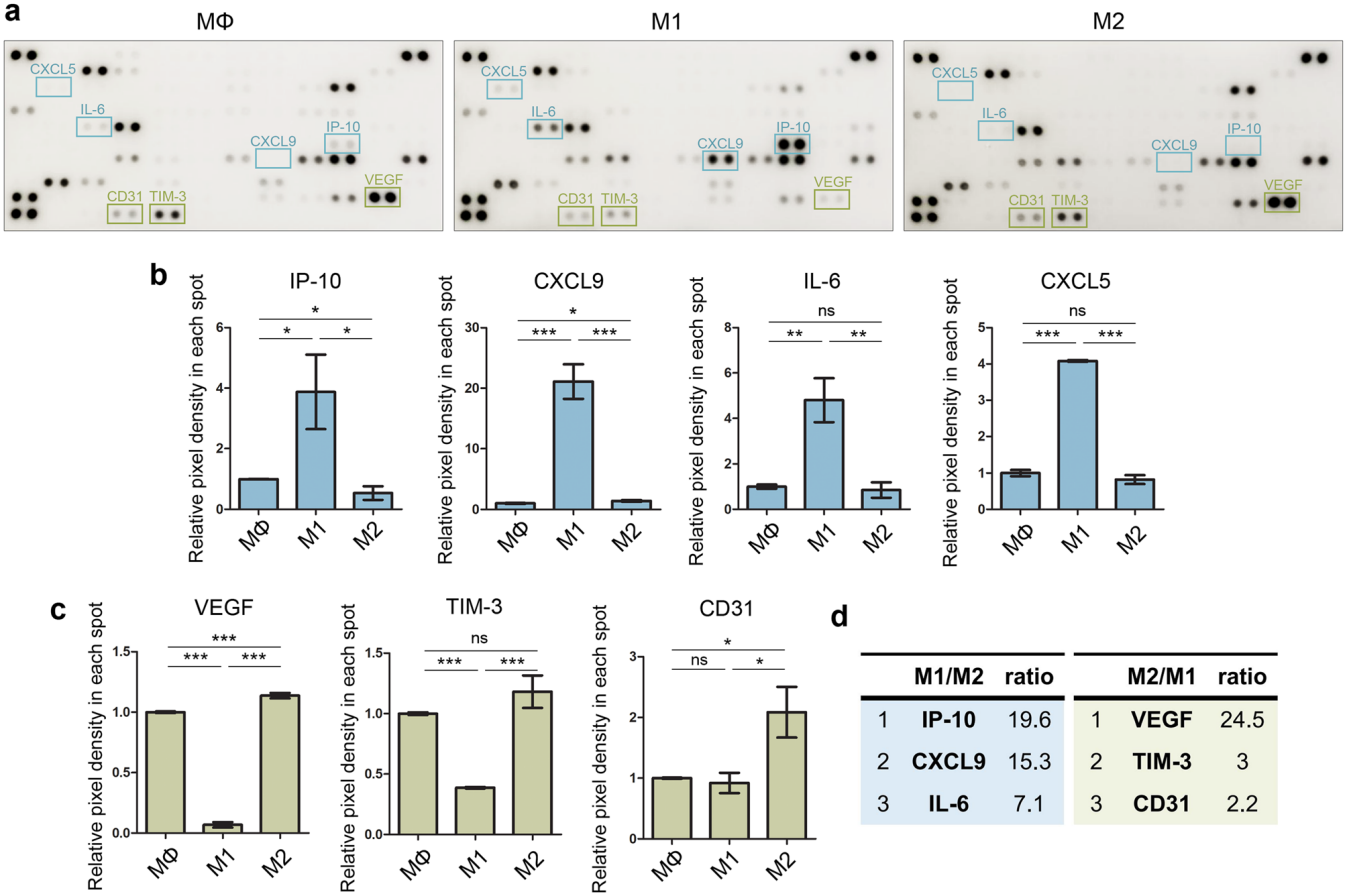
Confirmation of human cytokines in hiMel–macrophage coculture media. (**a**) Human cytokine array image of hiMel-macrophages (MΦ, M1, and M2) coculture media. Quantification of (**b**) proinflammatory markers and (**c**) proangiogenic markers. Data are expressed as the mean ± SEM. *p < 0.05, **p < 0.01, ***p < 0.001 indicate statistical significance. (**d**) Cytokines were ranked by M1/M2, M2/M1 ratio. *ns* non-significant, *MΦ* macrophage.

To identify cytokines secreted from macrophages that affect melanocyte pigmentation, a cytokine array was performed in the conditioned media of each hiMel and macrophage group (Fig. 4a). The levels of the inflammatory factors IP-10, CXCL9, IL-6, and CXCL5 in the hiMel-M1 group were higher than those in the hiMel-M2 group (Fig. 4b). The levels of the pro-angiogenesis factor TIM-3 was higher in the hiMel-M2 group than in the other groups. Moreover, the hiMel-M2 group showed increased secretion of the angiogenic factors VEGF and CD31 compared to in the hiMel-M1 group (Fig. 4c). The level of IP-10, which is secreted by M1 macrophages, was approximately 20-fold higher than that in other groups, and that of VEGF, which is secreted by M2 macrophages, was 25-fold higher than that in the hiMel-M1 group (Fig. 4d), suggesting that IP-10 secreted by M1 macrophages and VEGF secreted by M2 macrophages affected the pigmentation of hiMels in the coculture platform.

### Inhibition of VEGF reduced melanin production in cocultured hiMels with M2 macrophages

**Figure 5:**
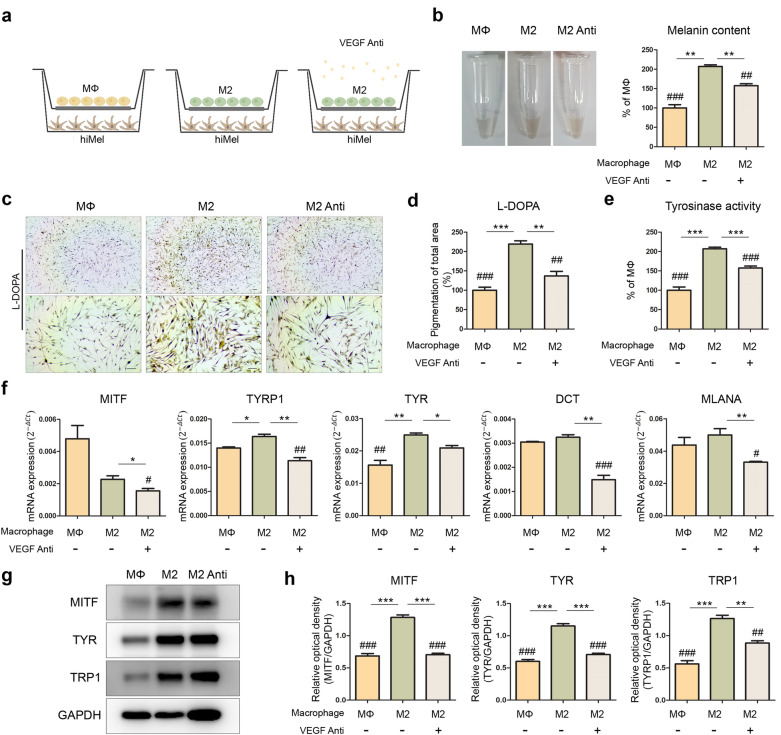
Neutralizing of hVEGF in hiMels cocultured with M2 macrophages. (**a**) Schematic diagram showing 5-day coculture of hiMels and M2 macrophages with or without 120 ng/mL human VEGF165 Antibody. (**b**) Melanin content of hiMels measured on day 5 coculture. (**c**) Pigmentation of cocultured hiMels with or without VEGF antibody by L-DOPA staining. Scale bar indicates 100 µm. (**d**) Quantitative measurements of L-DOPA staining area in cocultured hiMels by ImageJ software. (**e**) Quantification of tyrosinase activity in cocultured hiMels. (**f**) Quantitation of expression of pigmentation marker (MITF, TYRP1, TYR, DCT, and MLANA) in hiMels after 5 days of coculture. Relative gene expression values were calculated using the delta cycle-threshold (ΔCt) method. (**g**) Protein expression level of MITF, TYR, and TRP1 in hiMels. GAPDH was used as a loading control. (**h**) Quantification of western blotting using ImageJ software. *p < 0.05, **p < 0.01, ***p < 0.001 (*t*-test), ^#^*p* < 0.05, ^##^*p* < 0.01, ^###^*p* < 0.001 vs. M2 (one-way analysis of variance) indicate statistical significance. *MΦ* macrophage, *hiMels* human induced pluripotent stem cell (hiPSCs)-derived melanocytes, *VEGF anti* human VEGF165 antibody, *L-DOPA* 3,4-dihydroxyphenylalanine, *M2 anti* cocultured with M2 and VEGF antibody.

As shown in Fig. 4, VEGF was selected as a candidate affecting melanin biosynthesis in cocultured hiMels. To confirm this result, hiMels cocultured with M2 were treated with 120 ng/mL human VEGF165 antibody for 5 days of coculture (Fig. 5a). After coculture, the melanin content was measured in cocultured hiMels (Fig. 5b). hiMels cocultured with M2 showed upregulated melanin production compared to those cocultured with MΦ; however, this production was downregulated by neutralization of VEGF. VEGF neutralization also reduced melanogenesis in hiMels cocultured with M2 as confirmed by L-DOPA staining (Fig. 5c,d). Moreover, the tyrosinase activity assay of M2 cocultured hiMels with or without antibody showed similar levels of L-DOPA staining (Fig. 5e). Quantitative reverse transcription-PCR analysis of the *MITF*, *TYRP1*, *TYR*, *DCT*, and *MLANA* gene expression levels was performed in 5-day cocultured hiMels with or without VEGF antibody (Fig. 5f). In hiMels cocultured with M2, the expression of *TYRP1* and *TYR* mRNA was significantly increased compared to that in hiMels cocultured with MΦ, and hiMels cocultured with M2 and VEGF antibody showed significantly decreased *MITF*, *TYRP1*, *TYR*, *DCT*, and *MLANA* mRNA levels compared to without VEGF antibody. According to the western blotting results, the protein levels of the MITF, TYR, and TRP1 markers were similar to those in other experiments (Fig. 5g,h). These results suggest that VEGF secreted from M2 macrophages promoted melanin synthesis in the in vitro coculture system.

### Changes in melanin biosynthesis by VEGF in hiMels

**Figure 6:**
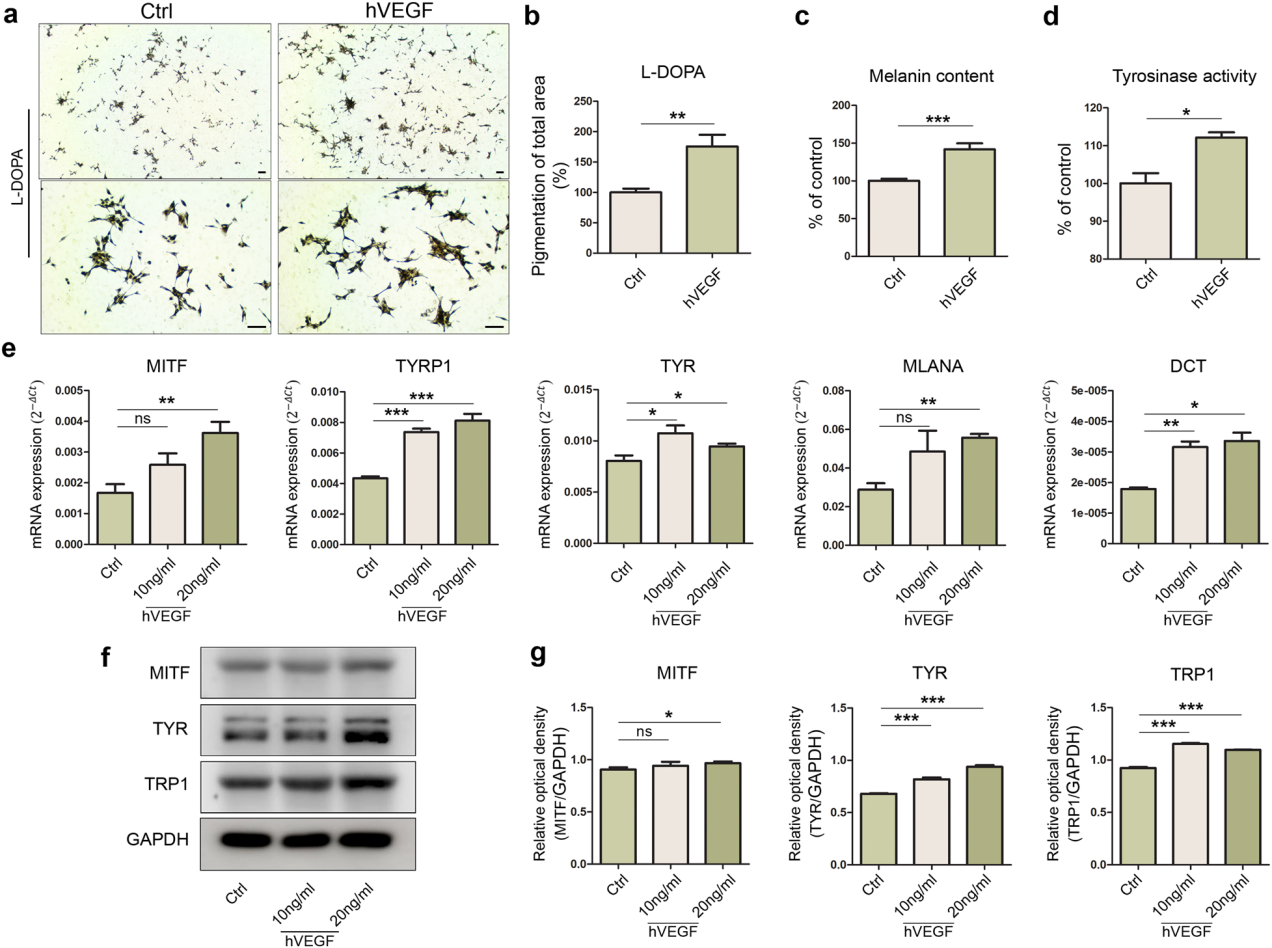
Effect of hVEGF on melanin synthesis in hiMels. (**a**) L-DOPA staining in hiMels following 20 ng/mL VEGF treatment for 72 h. (**b**) Quantification of total pigmentation area. (**c**) Changes in melanin content in hiMels treated with 20 ng/mL VEGF. (**d**) Quantification of tyrosinase activity in hiMels treated with 20 ng/mL VEGF. (**e**) Expression of pigmentation markers *MITF*, *TYRP1*, *TYR*, *MLANA*, and *DCT* upon treatment with VEGF. The relative gene expression values were calculated using the delta cycle-threshold (ΔCt) method. (**f**) Western blot analysis of MITF, TYR, and TRP1 in cell lysates. GAPDH was used as a loading control. (**g**) MITF, TYR, and TRP1 quantification using ImageJ software. Data are expressed as the mean ± SEM. *p < 0.05, **p < 0.01, ***p < 0.001 indicate statistical significance. *L-DOPA* 3,4-dihydroxyphenylalanine, *ns* non-significant.

To confirm the previous results, hiMels were treated VEGF. Before treatment, the CCK assay was performed to determine whether VEGF affected hiMel proliferation (Supplementary Fig. S2). VEGF (0–50 ng/mL) was not cytotoxic to hiMels after 72 h of treatment. Treatment with VEGF (20 ng/mL) activated melanin biosynthesis and melanogenesis according to L-DOPA staining (Fig. 6a). VEGF-treated hiMels showed higher percentage of pigmented areas than that seen with the control (Fig. 6b). The melanin content assay and tyrosinase activity assay also showed that melanin levels increased in VEGF-treated hiMels compared to in controls (Fig. 6c,d). Expression of mature melanocyte markers, such as *MITF*, *TYRP1*, *TYR*, and *MLAMA*, was upregulated by VEGF treatment, and most markers appeared to increase in a dose-dependent manner (Fig. 6e). Moreover, we confirmed that MITF, TYR, and TRP1 were more abundant in VEGF-treated iMels than in controls using western blot analysis (Fig. 6f,g). These results suggest that hiMels reacted with the selected cytokines and that the previous results were related to VEGF secreted by M2 macrophage types.

## Discussion

Skin pigmentation is a complication that typically occurs during scar inflammation after surgical procedures or burns^30^. When the skin is damaged, immune cells migrate to remodel the wound site; however, newly formed skin tissue may exhibit a different color compared to the original tissue^31^. Hyperpigmentation occurs because of high levels of inflammation in the scar^32^. This visible change in the skin during inflammation raises cosmetic and psychosocial concerns for the patient.

Macrophages play a critical role in innate immunity. When inflammation breaks because of scar formation, macrophages first migrate to the wound^14,17^. Upon reaching the wound site, M1 macrophages begin to secrete inflammatory cytokines such as IL-1β, IL-6, and IL-12 and remove bacteria or viruses that have entered the body^15,16^. During this process, the wounded area tends to become swollen, damaging surrounding cells. Once the foreign substances are removed, M2 macrophages migrate to the site and secrete anti-inflammatory cytokines such as IL-10, TGF-β1, and VEGF to remodel the surrounding tissues^33,34^. Skin pigmentation occurs during this process, which can sometimes persist for a long period^35^. The relationship between the wound healing process and scar pigmentation is not well-understood; therefore, continuous research on this topic is needed. Direct studies of melanocytes are complicated because of the lack of a source of these cells; thus, further research using a new cell source is required.

To overcome this issue, research using hiPSCs as a cell source has been actively conducted^36,37^. Importantly, iPSC technology can be used in studies requiring cells that are difficult to obtain, including melanocytes. Mica et al. differentiated hiPSCs into melanocytes through neural crest generation^7,28^. hiPSCs plated on Matrigel differentiated into neural crests, which were transferred onto fibronectin-coated plates as droplets, and Chir99021, BMP4, and EDN3 were used to generate mature melanocytes.

In this study, pigmentation of hiMels was visually confirmed in the cell pellets (Fig. 1c). Gene expression of melanocyte markers increased in hiMels, whereas pluripotency marker expression was decreased (Fig. 1e). We further confirmed that hiMels were positively stained using Fontana–Masson and L-DOPA staining, which are melanin-specific (Fig. 1f–h).

Travis et al. observed the process of hyperpigmentation during wound healing in swine models^31^. They reported that α-MSH, which has potent anti-inflammatory effects, increases pigmentation by regulating the expression of proinflammatory and anti-inflammatory cytokines^38^. This study suggests a close relationship between skin pigmentation and inflammation. Based on other studies, we attempted to confirm the effect of macrophages on pigmentation using a coculture platform. We differentiated M1 and M2 macrophages from THP-1 monocytes to mimic the human immune system in vitro. THP-1 monocytes were differentiated into MΦ and then polarized into M1 and M2 macrophages. We confirmed the gene expression levels of macrophage markers. The levels of inflammatory markers, such as *CXCL10*, *IL-6*, *IL-1β*, and *IL-12*, were increased in M1 macrophages (Fig. 2b). In contrast, the expression of wound healing markers, such as *CD163*, *CD206*, *IL-10*, *and VEGF*, was higher in M2 macrophages (Fig. 2c). Expression of the M1 cytokine IL-6 and M2 cytokine TGF-β was also confirmed (Fig. 2d). We used an in vitro coculture system to investigate the interaction between macrophages and pigmentation. After 5 days of coculture, melanin levels were low in the inflammatory environment and high in the wound healing environment (Fig. 3b). A similar tendency was observed with L-DOPA staining (Fig. 3d). The levels of the pigmented area increased in M2 cocultured hiMels, whereas M1 cocultured hiMels showed lower levels compared to M0 cocultured hiMels (Fig. 3e). We also confirmed the gene expression levels of pigmentation markers (Fig. 3f).The gene expression levels of pigmentation markers were significantly decreased in the inflammatory environment and increased in the wound healing environment induced by different macrophage types. Based on these results, inflammation reduces melanocyte pigmentation, whereas the tissue repair environment promotes melanocyte pigmentation.

To identify the specific cytokines affecting pigmentation in each environment, a human cytokine array was performed with the coculture media. Inflammatory cytokines were generally detected in the hiMels-M1 group and IP-10 levels were significantly increased (Fig. 4b). High levels of cytokines related to tissue repair were detected in the hiMel-M2 group (Fig. 4d). The VEGF level was higher in the culture supernatant of the hiMel-M2 group than in the hiMel-M1 group. We listed M1/M2 and M2/M1 ratios in descending order (Fig. 4e). VEGF, which showed the greatest difference, was selected for neutralization in the coculture system to confirm the pigmentation changes induced by macrophages. As a result, melanogenesis was significantly reduced by the VEGF antibody in hiMels cocultured with M2 (Fig. 5). In addition, we directly treated hiMels with VEGF and verified the changes in melanin production. VEGF treatment increased pigmentation in the melanin content assay and tyrosinase activity assay (Fig. 6c,d). Gene expression and protein expression of pigmentation markers was altered, similar to the results of coculturing cells in VEGF-treated hiMels (Fig. 6e–g). Previous studies have shown that tyrosinase protein size can be either glycosylated or deglycosylated^39,40^; thus, in the western blot results, we observed two different forms of tyrosinase bands (Figs. 5g, 6f, respectively). The deglycosylated form of tyrosinase was detected at 60 kDa (Fig. 5g), whereas the glycosylated form appeared at 80–90 kDa (Fig. 5g). These results are thought to be because of the distinct differentiation efficiency of iPSCs and iMels. It is hypothesized that even if the differentiation process or the harvest timepoints are the same, the degree of iMel maturity can be different. Moreover, it can be also speculated that tyrosinase was deglycosylated during coculture with macrophages.

The effects of VEGF on melanocytes have been reported in several studies. Zhu et al. showed that VEGF enhanced tyrosinase activity and melanin production^27^. Regazzetti et al. attempted to confirm the effect of endothelial cells on melanocytes because they observed that vascularization affected skin pigmentation in patients with benign vascular skin lesions^41^. By coculturing human dermal microvascular endothelial cells with human melanocytes, the authors suggested that VEGF is involved in melanogenesis and melanocyte proliferation. Although the effect of VEGF secreted by M2 macrophages on melanocytes or pigmentation has not been evaluated, our study suggests that this secretion affects melanocyte activity or hyperpigmentation during inflammation in the skin tissue.

There were several limitations to our study. Our in vitro system does not fully mimic the in vivo environment, as the inflammation process typically requires both M1 and M2 in the same environment. Further studies of the effect of VEGF as a key cytokine and confirmation of the related pathways causing melanocyte pigmentation through these factors are needed. Macrophages produce many other factors that regulate melanin synthesis. Slominiski et al. confirmed that pro-opiomelanocortin produced by macrophages upregulates melanogenesis^42,43^. Additionally, inflammatory factors such as TNF, IL-1β, and IFN-γ^23^ were reported to inhibit melanin synthesis, whereas cytokines such as IL-18^25^ and GM-CSF upregulate melanogenesis^20^. Confirmation of these proteins using our platform would be interesting as further studies. We measured melanin content as the optical density at 405 nm^44,45^; however, several studies have suggested that this is not a direct measurement of the actual amount of melanin. More accurate measurements of the melanin content using high-performance liquid chromatography or electron paramagnetic resonance methods are needed^46–48^. We hope to confirm our results using these methods in future studies.

In conclusion, we showed that M1 and M2 macrophages decreased and increased melanin synthesis in melanocytes, respectively. VEGF expression showed the greatest increase in M2 coculture culture media. Thus, melanin production may be suppressed by cytokines secreted by M1 macrophages during the early stages of inflammation. The increased expression of VEGF by M2 macrophages may increase pigmentation and induce hyperpigmentation in the scarred area during the tissue repair process. Therefore, VEGF is a potential target for regulating skin pigmentation.

## Methods

### hiPSC culture

All hiPSC lines used in this study were generated using peripheral blood mononuclear cells. Reprogramming and characterization were performed as previously described^49,50^. The cells were maintained in vitronectin-coated dishes (Thermo Fisher Scientific, Waltham, MA, USA), and the media were changed daily with fresh Essential 8 (E8) medium (Thermo Fisher Scientific).

### Melanocyte differentiation from hiPSCs

Melanocytes were differentiated as previously described^7,28^. Briefly, 5 × 10^4^ human hiPSCs were plated on a Matrigel-coated 12-well in E8 medium containing Rho-associated protein kinase (ROCK inhibitor, 10 mM; Y-27632, PeproTech, Rocky Hill, NJ, USA). The cells were allowed to reach 70% confluence over 2–3 days with daily media changes. Differentiation was initiated by switching the medium to knockout serum replacement medium supplemented with LDN193189 (500 nM; PeproTech) and SB431542 (10 mM; Tocris, Bristol, UK). Treatment with CHIR99021 (3 mM; PeproTech) was started on day 2 of differentiation, and LDN193189 and SB431542 were withdrawn on days 3 and 4, respectively. On day 4, knockout serum replacement medium was gradually replaced with increasing amounts of N2 medium. The cells were additionally treated with human recombinant bone morphogenetic protein 4 (BMP4, 25 ng/mL; R&D Systems, Minneapolis, MN, USA) and endothelin 3 (EDN3, 100 nM; Tocris) beginning on day 5 of differentiation. The cells were collected on day 8 for passaging, dissociated, and plated on fibronectin-coated plates in full Mel medium containing CHIR99021 (3 mM), BMP4 (25 ng/mL), and EDN3 (100 nM). The media were changed on alternate days and the cells were passaged weekly for maintenance and expansion. For cytokine treatment, 1 × 10^5^ melanocytes were plated into 12-well dishes. After overnight incubation, the medium was changed to differentiation medium containing the indicated concentrations of VEGF (PeproTech).

Human epidermal melanocytes (NHEMs, dark donor, c2025c, Thermo Fisher Scientific) were maintained in Medium 254 (Thermo Fisher Scientific) with human melanocyte growth supplement (Thermo Fisher Scientific).

### Polarization of macrophages into M1 and M2

The human monocytic leukemia cell line, THP1 was a kind gift from Dr. Youngkyun Kim (Seoul National University, Seoul, South Korea). Human monocytes THP-1 cells were maintained in Roswell Park Memorial Institute medium (RPMI 1640, Thermo Fisher Scientific) containing 10% heat-inactivated fetal bovine serum (Thermo Fisher Scientific), HEPES (10 mM; Thermo Fisher Scientific), and β-mercaptoethanol (50 pM; Thermo Fisher Scientific). THP-1 monocytes were differentiated into macrophages by 24-h incubation with phorbol 12-myristate 13-acetate (150 nM; Sigma Aldrich, St. Louis, MO, USA), followed by 24-h incubation in RPMI medium. Macrophages were polarized into M1 macrophages with IFN-γ (100 ng/mL; R&D Systems) and lipopolysaccharide (10 ng/mL; Sigma) for a further 24 h. Macrophage M2 polarization was achieved by incubation with IL-4 (20 ng/mL; R&D Systems) and IL-13 (20 ng/mL; R&D Systems) for 24 h as previously reported^29^.

### Coculture of hiMels with M1 and M2 macrophages

For coculture experiments, 4 × 10^5^ THP-1 monocytes were differentiated and polarized in 12-well Transwell™ inserts (#3460, Corning, Inc., Corning, NY, USA). hiMels were plated onto fibronectin (Roche, Basel, Switzerland)-coated 12-well dishes at a density of 1 × 10^5^ cells per well. After fully differentiating each cell, coculture was performed by inserting the macrophage-containing inserts into the hiMel-containing 12-well dishes. The cells were incubated in a combination of media comprised of 70% full Mel medium and 30% RPMI medium supplemented with CHIR99021 (3 mM), BMP4 (25 ng/mL), and EDN3 (100 nM), with or without human VEGF165 antibody (120 ng/mL; AF-293-SP, R&D Systems) at 37 °C and 5% CO_2_. After 24, 72, and 120 h of coculture, the upper inserts were removed, and the supernatants were collected for cytokine analysis. The cocultured hiMels were harvested for further analysis. All experiments were performed in triplicate.

### Transmission electron microscopy

Cell pellets were fixed in 2.5% glutaraldehyde in phosphate-buffered saline (PBS; pH 7.2) at 4 °C and then conventionally embedded and sectioned. The slices were viewed using a transmission electron microscope (JEOL-JEM 1010, JEOL Co., Ltd., Tokyo, Japan).

### Polymerase chain reaction

Total mRNA was extracted using TRIzol reagent (Thermo Fisher Scientific). cDNA was synthesized using a RevertAid First Strand cDNA Synthesis Kit (Thermo Fisher Scientific). Quantitative real-time PCR was performed using SYBR Green Mix and a LightCycler 480 Instrument II (Roche). The results were calculated using the LightCycler (Roche Diagnostics). Relative gene expression was calculated using the delta cycle-threshold (ΔCt) method, and the ΔCt values were converted to ratios^51,52^. The primer sequences are presented in Supplementary Table S1.

### L-DOPA staining

Cells or tissue sections were fixed with 4% paraformaldehyde for 30 min. The cells were washed twice with PBS and incubated with 0.1% L-DOPA (3,4-dihydroxyphenylalanine; Sigma) at 37 °C for 4 h. Stained cells were observed under a bright-field microscope.

### Fontana–Masson staining

Fontana–Masson staining kits (ab150669, Abcam, Cambridge, UK) were used to visualize the melanocytes. Briefly, the cells were fixed with 4% paraformaldehyde and then incubated in ammoniacal silver solution until the cells turned brownish in color (~ 45 min). The cells were sequentially treated with 0.2% gold chloride solution for 30 s and in 5% sodium thiosulfate solution for 2 min. The samples were mounted, and staining was confirmed using a bright-field microscope.

### Immunocytochemistry

The cells were washed with 1 × PBS and fixed with 4% paraformaldehyde for 30 min at 25 °C. Next, 0.1% Triton X-100 was added and incubated at 25 °C for 10 min. Thereafter, we added PBS containing 2% bovine serum albumin (BSA) for 30 min for blocking. Anti-TRP1 antibody (ab235447; Abcam) was diluted at a 1/200 ratio in PBS containing 2% BSA for 2 h at 25 °C. Anti-CD68 antibody (ab955; Abcam), anti-TGF-β antibody (ab27969; Abcam), and anti-IL-6 antibody (ab9324; Abcam) were diluted in a 1/200 ratio in PBS containing 2% BSA overnight at 4 °C. Alexa Fluor^®^594-conjugated goat anti-rabbit IgG (H + L) antibody (A11037, Molecular Probes, Eugene, OR, USA) and Alexa Fluor^®^488-conjugated goat anti-mouse IgG (H + L) antibody (A11029, Molecular Probes) were diluted in PBS containing 2% BSA and incubated for 1 h at 25 °C. Finally, 4,6-diamidino-2-phenylindole (DAPI; 10236276001; Roche) was applied at 25 °C for 10 min. The cells were washed with PBS and images were obtained using an upright fluorescence microscope (Axio Imager.M2; Carl Zeiss, Oberkochen, Germany) for iMel and confocal laser scanning microscope (LSM800 w/Airyscan; Carl Zeiss) for macrophages.

### Tyrosinase activity assay

Cells were washed with PBS and lysed with radio-immunoprecipitation assay buffer (R0278, Sigma) supplemented with phosphatase inhibitor cocktail set IV (1:50, 524628; Merck Millipore, Billerica, MA, USA) and PMSF protease inhibitors (1 nM). Cell lysates were centrifuged at 14,000×*g* for 30 min at 4 °C, and a Bradford assay was performed. A protein suspension (30 µL) containing 30 µg total protein and 100 µL 0.1% L-DOPA in PBS was mixed in a 96-well plate. After incubation at 37℃ for 1 h, dopachrome formation was measured at 475 nm. Tyrosinase activity was calculated as the percentage of the control.

### Melanin content assay

Total melanin was measured in the cell pellets by dissolving them in 1 N NaOH for 30 min at 25 °C. Solubilized melanin was quantified at 405 nm. The melanin content was calculated as a percentage compared to the control.

### Western blot analysis

The protein was extracted from the cells as described for the tyrosinase activity assay. Proteins were separated using 8% sodium dodecyl sulfate-polyacrylamide gel electrophoresis and transferred onto nitrocellulose membranes. The membranes were blocked with PBS containing 0.1% Tween 20 (PBST) and 3% BSA for 1 h and then incubated with an anti-MITF antibody (1:1000, ab12039; Abcam), anti-tyrosinase antibody (1:500, sc-20035; Santa Cruz Biotechnology, Dallas, TX, USA), and anti-TRP1 antibody (1:1000, ab235447; Abcam) at 4 °C. The next day, membranes were washed 3 times with 0.1% PBST and incubated for 1 h with the respective secondary antibodies. After 4 washes with 0.1% PBST, the protein-of-interest was detected using a Bio-Image Analysis System (Amersham Imager 600; Fuji Photo Film Co., Ltd., Tokyo, Japan).

### Inflammatory cytokine array

A human XL cytokine array kit (ARY022B, R&D Systems) was used according to the manufacturer’s protocol. Briefly, the membranes were blocked for 1 h and then incubated with coculture media overnight at 4 °C. The detection antibody cocktail was diluted in 1 × array buffer and incubated for 1 h at 25 °C. Images were obtained using the Bio-Image Analysis System (Amersham Imager 600). The intensity of the spots was quantified using ImageJ software (National Institute of Health, Bethesda, MD, USA).

### Cell viability assay

To confirm the viability of the melanocytes, we performed a cell counting Kit-8 assay (CCK-8; CK04-13, Dojindo Molecular Technologies, Kumamoto, Japan). The culture media supernatant was transferred to a 96-well plate after 72 h, and 10 μL CCK-8 solution was added to each well. Absorbance was measured at 450 nm using a microplate reader.

### Statistical analysis

All experiments were repeated at least three times. Statistical analyses were performed using GraphPad Prism 5 software (GraphPad, Inc., La Jolla, CA, USA). The results are presented as the mean and standard error of the mean. Error bars represent the standard error of the mean. Differences between groups were examined for statistical significance using Student’s *t* test. A *t* test was used to analyze nonparametric quantitative datasets, and a one-tailed p-value was calculated. One-way analysis of variance, followed by Dunnett’s multiple comparison test, was used for several analyses (*p < 0.05, **p < 0.01, and ***p < 0.001 indicate statistical significance).

## Supplementary Information

Supplementary Information.
