# Supplementary material for: Preferential stimulation of melanocytes by M2 macrophages to produce melanin through vascular endothelial growth factor
Source: Sci Rep. 2022 Apr 19;12:6416. doi: 10.1038/s41598-022-08163-7 (PMC9019043; doi:10.1038/s41598-022-08163-7)
Supplement: Supplementary file 1 — Supplementary Information. [file 41598_2022_8163_MOESM1_ESM.docx]

Preferential stimulation of melanocytes by M2 macrophages to produce melanin through vascular endothelial growth factor

Heeju Han^1,2^, Yena Kim^1^, Hyunkyung Mo^1,2^, Si Hwa Choi^1,2^, Kijun Lee^1,2^, Yeri Alice Rim^1^, and Ji Hyeon Ju^1,3^

^1^Catholic iPSC Research Center (CiRC), CiSTEM Laboratory, Department of Biomedicine & Health Sciences, College of Medicine, The Catholic University of Korea, Seoul 06591, South Korea

^2^Department of Medical Life Sciences and Department of Biomedicine & Health Sciences, College of Medicine, The Catholic University of Korea, Seoul 06591, South Korea

^3^Division of Rheumatology, Department of Internal Medicine, College of Medicine, Seoul St. Mary's Hospital, The Catholic University of Korea, Seoul 06591, South Korea

**Corresponding author:**

Ji Hyeon Ju, MD, PhD

505 Banpo-dong, Seocho-gu, Division of Rheumatology, Department of Internal Medicine, Seoul St. Mary’s Hospital, College of Medicine, The Catholic University of Korea, Seoul 137-040, South Korea (Tel: 82-2-2258-6893, Fax: 82-2-3476-2274, E-mail address: [juji@catholic.ac.kr](mailto:juji@catholic.ac.kr))

**AUTHOR CONTRIBUTIONS**

H.H. and J.H.J. designed the study. K.L. contributed to designed the study. H.H. collated the data, analyzed the data, and wrote the initial draft of the manuscript. S.C. and H.M. contributed to the data analysis. Y.K., Y.A.R., and J.H.J. contributed to the data analysis and to drafting the manuscript. All authors have read and approved the final submitted manuscript.
